# Supplementary material for: Reconciling Mining with the Conservation of Cave Biodiversity: A Quantitative Baseline to Help Establish Conservation Priorities
Source: PLoS One. 2016 Dec 20;11(12):e0168348. doi: 10.1371/journal.pone.0168348 (PMC5173368; doi:10.1371/journal.pone.0168348)
Supplement: S1 Dataset — (ZIP) [file pone.0168348.s002.zip › Taxa/Serra Norte/SN_2013/N5S/Lista N5S-72.pdf]

CAVIDADE N5S-0072

Classe	Ordem	Fam/Outros	Gên/Outros	Espécie	S-U	U-S	Zon.
Arachnida	Acari	Ixodidae		jovem	X		E
Arachnida	Acari	Mesostigmata		sp.43	X		E
Arachnida	Acari	Veigaiidae		sp.1	X		E
Arachnida	Amblypygi	Phrynidae	*Heterophrynus*	*longicornis*	5	1	E/P
Arachnida	Araneae	Ctenidae		jovem	3		E/P
Arachnida	Araneae	Oonopidae	*Capitato*	sp.1		X	P
Arachnida	Araneae	Paratropididae		sp.1	1		E
Arachnida	Araneae	Pholcidae	*Mesabolivar*	*aurantiacus*	X	X	E/P
Arachnida	Araneae	Pisauridae		jovem	1	3	E/P
Arachnida	Araneae	Scytodidae	*Scytodes*	sp.1		1	P
Arachnida	Araneae	Theraphosidae		jovem		1	P
Arachnida	Araneae	Theridiosomatidae	*Plato*	sp.1	X	X	P
Arachnida	Araneae	Trechaleidae	*Rhoicinus*	sp.1	1		E
Arachnida	Opiliones	Sclerosomatidae	*Prionostema*	sp.1		1	E
Chilopoda	Geophilomorpha	Geophilidae		sp.1		1	P
Diplopoda	Polydesmida	Chelodesmidae		jovem	1		E
Diplopoda	Polydesmida	Cyrtodesmidae		jovem	X		E
Diplopoda	Polydesmida	Pyrgodesmidae		jovem	X		E
Diplopoda	Siphonophorida	Siphonophoridae		jovem	X		E
Entognatha	Collembola	Entomobryidae		sp.1		X	P
Entognatha	Collembola	Entomobryidae		sp.10	X		E
Entognatha	Collembola	Paronellidae		sp.1	X	X	E/P
Insecta	Blattodea			jovem	2	1	P
Insecta	Coleoptera	Dytiscidae		sp.9	X		E
Insecta	Coleoptera	Hydrophilidae		sp.2	X		E
Insecta	Coleoptera	Scydmaenidae		sp.3	X		P
Insecta	Coleoptera	Staphylinidae	Pselaphinae	sp.2		X	P
Insecta	Coleoptera	Staphylinidae	Pselaphinae	sp.4	X		E
Insecta	Coleoptera	Staphylinidae	Pselaphinae	sp.14	X		E
Insecta	Coleoptera	Staphylinidae		sp.4	X		E/P
Insecta	Coleoptera	Staphylinidae		sp.11		X	P
Insecta	Coleoptera	Staphylinidae		sp.19	X		E
Insecta	Coleoptera	Staphylinidae		sp.20	X		E
Insecta	Diptera	Cecidomyiidae	Cecidomyiinae	sp.	X		P
Insecta	Diptera	Conopidae	*Stylogaster*	sp.		X	E
Insecta	Diptera	Empidiidae		sp.	X		E
Insecta	Diptera	Limoniidae		sp.		X	P
Insecta	Diptera	Psychodidae	Phlebotominae	sp.	X		P
Insecta	Diptera	Sciaridae		sp.	X		E
Insecta	Diptera	Tipulidae	Tipulinae	sp.	X	X	E/P
Insecta	Ephemeroptera	Leptophlebiidae		jovem		X	E
Insecta	Heteroptera	Dipsocoroidea		sp.	X		E
Insecta	Heteroptera	Hebridae	*Hebrus*	sp.1	X		E
Insecta	Heteroptera	Veliidae	*Paravelia*	sp.		X	E
Insecta	Heteroptera	Veliidae	*Rhagovelia*	sp.	X		E
Insecta	Homoptera	Cixiidae		jovem	X		E
Insecta	Hymenoptera	Formicidae	*Brachymyrmex*	sp.1	X	X	E/P
Insecta	Hymenoptera	Formicidae	*Crematogaster*	sp.1		X	P
Insecta	Hymenoptera	Formicidae	*Eurhopalothrix*	sp.1		X	P

Insecta	Hymenoptera	Formicidae	*Hypoponera*	sp.1	X		E
Insecta	Hymenoptera	Formicidae	*Pachycondyla*	sp.1	X		E
Insecta	Hymenoptera	Formicidae	*Pachycondyla*	sp.2		X	P
Insecta	Hymenoptera	Formicidae	*Paratrechina*	sp.1	X	X	E/P
Insecta	Hymenoptera	Formicidae	*Paratrechina*	sp.2		X	P
Insecta	Isoptera	Termitidae	*Diversitermes*	sp.	X		E
Insecta	Isoptera	Termitidae	*Nasutitermes*	sp.	X	X	E/P
Insecta	Lepidoptera	Galacticidae		sp.	X		E
Insecta	Lepidoptera	Noctuoidea		sp.5	2		P
Insecta	Lepidoptera	Tineoidea		sp.1	X		P
Insecta	Lepidoptera	Tineoidea		sp.3		X	E
Insecta	Orthoptera	Phalangopsidae	*Paraclodes*	sp.	9	1	E
Insecta	Orthoptera	Phalangopsidae	*Phalangopsis*	sp.	9	16	E/P
Insecta	Psocoptera			jovem	X		E
Insecta	Thysanura	Nicoletiidae		jovem	X		P
Gastropoda	Pulmonata	Systrophiidae	*Happia*	sp.	X		E
Malacostraca	Isopoda	Platyarthridae		sp.1	X		E
Turbellaria				sp.4	X		E
Amphibia	Anura	Strabomantidae	*Pristimantis*	cf. *fenestratus*	5		E/P
Mammalia	Chiroptera	Phyllostomidae	*Carollia*	*perspicillata*		1	E
Mammalia	Rodentia	Cricetidae	*Rhipidomys*	sp.	1		E
Reptilia	Squamata	Sauria		sp.	1		E
